# Drug-induced Sudden Death: A Scoping Review

**DOI:** 10.2174/1574886317666220525115232

**Published:** 2023-04-25

**Authors:** Kristopher Amaro-Hosey, Xavier Castells, Lidia Blanco-Silvente, Pablo Loma-Osorio, Dolors Capellà

**Affiliations:** 1 Clinical Pharmacology Service, Hospital de la Santa Creu i Sant Pau, Barcelona, Spain;; 2 Department of Pharmacology, Therapeutics and Toxicology, Faculty of Medicine, Autonomous University of Barcelona, Barcelona, Spain;; 3 TransLab Research Group, Department of Medical Sciences, Faculty of Medicine, University of Girona, Girona, Spain;; 4 Department of Medical Sciences, Faculty of Medicine, Cardiology Service, Hospital Universitari Josep Trueta, University of Girona, Girona, Spain

**Keywords:** Sudden cardiac death, drug-induced, side effect, adverse drug reactions, epidemiological studies, scopes review

## Abstract

**Background:**

The risk of sudden cardiac death (SCD) can be increased with the use of drugs. However, it has been described heterogeneously in the literature.

**Objective:**

This study aims to systematically review epidemiological studies dealing with drug-induced sudden death, describe their methodologies, and summarize the results found.

**Methods:**

A scoping review has been carried out using Medline electronic database. The search was limited up to 2020. Epidemiological studies were included, and case reports or case series were excluded.

**Results:**

Out of 3,114 potential articles, 74 were included. Most studies originated from North America (40.5%) or Europe (39.2%). Case-control (47.3%) or cohort (40.5%) studies were the most common designs. The data for outcomes and exposure were retrieved mainly from administrative databases (37.8%) or medical charts/hospital discharge reports (32.4%), but most studies used several sources of information. A composite variable of sudden death or SCD, mainly with ventricular arrhythmia, was the most frequently used endpoint. Only 18.9% of the studies included autopsy results to confirm the death. Psychotropic drugs were the most frequently studied. An increased risk of different outcomes for typical antipsychotics, tricyclic antidepressants, domperidone, and antiepileptics is suggested.

**Conclusion:**

The methodologies used were highly heterogeneous, and the results were, in general, not conclusive. An improvement of the methodologies is needed to achieve a conclusion regarding the risk of SCD associated with drug use.

## 1 INTRODUCTION

Sudden cardiac death (SCD) describes the unexpected natural death due to a cardiac etiology in a brief time period, generally in the first hour of the onset of the symptoms (even if some definitions extend it to the first 24 h) or in the absence of witnesses when the deceased has been seen in good condition less than 24 hours before the death, in a person without any previous condition that seems fatal [1-3]. Some patients die instantly, but most experience some prodromal symptoms [1]. Prodromal symptoms are often unspecific, and even suspected symptoms of ischemia (chest pain), tachyarrhythmia (palpitations) or congestive heart disease (dyspnea) can only be considered suggestive [2].

SCD represents 50% of cardiovascular mortality in developed societies [2]. Prevalence is estimated between 300,000 and 400,000 annual cases in the USA, and the incidence is estimated to be 100 cases per 100,000 inhabitants in the general population [3]. The causes of SCD are complex and can be viewed as an interaction between structural heart abnormalities, transient functional disturbances, and specific electrophysiological events responsible for fatal arrhythmias. It is partly explained by established risk factors, such as ischemic heart disease, heart failure, and electrolyte imbalance [4].

The risk of SCD can be increased with the use of some pharmacological treatments. Drugs that have been involved in SCD mainly include those used to treat non-heart diseases since these drugs may alter cardiac depolarization or repolarization and thus increase the risk of fatal cardiac arrhythmias (such as ventricular fibrillation) [5-7]. The importance of SCD associated with the use of drugs is remarkable, as evidenced. In fact, ventricular arrhythmias (VA) have been one of the most common causes of withdrawal from the marketing of drugs or the restriction of their conditions of use. In recent decades, this has been associated with the use of terfenadine [8, 9], thioridazine, sertindole [9], cisapride [10], and more recently, citalopram [11] and azithromycin [12], among other drugs. However, despite its importance, sudden death cases attributed to drugs have been described heterogeneously in the literature, using observational methods, case-control or cohort designs, and even clinical trials. In order to summarize the evidence on SCD related to drug use and the methodological characteristics of the studies performed as well as their results, a scoping review has been carried out.

## 2 METHODS

### 2.1 Systematic Literature Search

A systematic literature review was conducted in accordance with the PRISMA Extension for Scoping Reviews [13], using the Medline electronic database to search for studies that investigated associations between sudden death and drugs from inception to 2020, December 31^st^.

The search strategy was selected with an agreement using the following terms: “(death, sudden/etiology [MeSH Terms] OR death, sudden, cardiac/etiology [MeSH Terms]) AND (epidemiologic studies [MeSH Terms] OR epidemiological studies)”. References of the articles assessed for eligibility were also reviewed and included if they met inclusion criteria.

### 2.2 Inclusion and Exclusion Criteria

Any type of epidemiological study that evaluated the risk of sudden death associated with drug use was included. Case reports or case-series designs were excluded. No language, age, population or other search restrictions were applied.

### 2.3 Screening and Data Extraction

All articles were screened independently by 2 authors (KA and DC) to identify relevant studies based on titles and abstracts. The full texts of potentially relevant papers were also reviewed if study relevance could not be determined from the titles and abstracts.

Data were extracted independently for all included studies using a standardized data collection form defined and agreed upon previously. Data extracted were article identification, geographical area, study design, source of outcome data, source of exposure data, study period, outcome variable, and drug or group of drugs investigated, inclusion and exclusion criteria, confounding adjustment methods, study population, and results. A third author (LB) was involved in the study selection and the data extraction in case of disagreement.

### 2.4 Synthesis of Results

Data were analyzed using descriptive statistics. Based on the PRISMA-ScR recommendations, we agreed not to perform a critical appraisal analysis of the studies included [13].

## 3 RESULTS

In total, using both cited search strategies, 3,114 studies were retrieved from the Medline database. After excluding duplicates and non-relevant abstracts, 99 studies were considered relevant for eligibility, and finally, 74 studies [14-87] were included in the scoping review (see **S1** Table for characteristics of included studies). Fig. (**1**) shows the study flow chart.

### 3.1 General Characteristics of Included Articles

The main characteristics of the included studies are shown in Table **1**. The first study was published in 1992. Studies have increased from 12 (16.9%) in 1990-1999 to 37 (50%) in 2010-2020. Most of the studies were conducted in North America or Europe (40.5% and 39.2%). The proportion of studies performed in Europe increased from 25.0% in 1990-99 to 40.5% in 2010-20.

### 3.2 Methodology of the Studies

Case-control (47.3%) and cohort (40.5%) were the most used designs. Case studies were only used in 7 (9.5%) studies and often (4 studies) as a complementary design. Three studies performed a meta-analysis of observational studies, another 2 were systematic reviews of clinical trials with or without observational studies, and 2 used data collected during the clinical trials.

Most studies (78.4%) adjusted for confounding factors, mainly by multivariate modeling. This proportion has increased from 58.3% in the first decade to 81.1% in the last decade.

The source of information most commonly used to identify patients and collect outcome information and clinical data was an administrative database (a database designed for administrative purposes) in 28 studies (37.8%) or an epidemiological database (a database designed to perform epidemiological studies) in 14 (19.9%). Other sources of information were the medical charts/hospital discharge report in 24 (32.4%), death certificates in 15 (20.3%), autopsies in 14 (18.9%) or patient’s registry in 11 (14.9%) studies. The type of the database has changed over time. While the proportion of studies using an administrative database has decreased from 41.7% in 1990-1999 to 32.4% in 2000-2020, and those using an epidemiological database increased from 8.3% to 18.9%, respectively. Most of the studies used several sources to complete the information. In fact, only 30 (40.5%) studies used a unique information source (12 studies used an administrative database, 8 an epidemiological database, 3 a patient’s registry, 2 data collected during a clinical trial, 2 from the hospital medical record, 2 from the autopsy, and one from the mortality statistics). Administrative databases also used information from death certificates (7 studies), medical chart/hospital discharges (6 studies), mortality registers (4 studies), and/or patient’s registry (2 studies). Four studies performed with the information contained in an epidemiological database also used death certificates (3 studies) and/or contact with the physician, medical chart, and cohort (one study each). Moreover, studies using a medical chart/hospital discharge record or an autopsy or a death certificate were very rarely done without other sources of information. Studies using patient’s registries completed the information with medical charts (4 studies) and/or autopsies (3 studies). In 3 studies, relatives were contacted, and in another 2, the physician was requested to provide complementary information. Study characteristics are summarized in Table **1**.

### 3.3 Outcome Definition

The most frequently used outcome was a composite of sudden death (SD) or SCD, mainly with VA (26 studies, 35.1%). In 18 (24.3%) studies, the outcome variable was SCD, and in another 14 (18.9%), the outcome was SD. SUDEP (sudden unexpected death in epilepsy) was the outcome in 6 (8.1%) studies and SIDS (sudden infant death syndrome) in another 4 (5.4%). Other outcome variables less frequently used were cardiac arrest (CA), global or cardiovascular mortality or VA alone (Table **1**). An expert or a committee validated the outcome variable in 12 studies (16.2%).

### 3.4 Drugs Investigated

Psychotropic drugs were the most frequently studied (26 studies), mainly antipsychotics, as a group (10 studies, in one with antidepressants) or as individual agents (4 studies). Antidepressants accounted for 9 studies (5 as a group, including one with antipsychotics and 4 as different individual agents) and attention deficit hyperactivity disorder stimulants accounted for 4 additional studies. The following groups, in order of frequency, were cardiovascular drugs in 8 studies (antiarrhythmics in 4 studies and antihypertensives in 4 studies), propulsive (8 studies, mainly domperidone), antiepileptics (6 studies), different antibiotics (5 studies, mainly macrolides), diphtheria-tetanus-pertussis (DTP) vaccine (4 studies), non-antiarrhythmic QT modifiers (4 studies), antihistamines (3 studies), COX-2 inhibitors (2 studies), second-generation sulfonylureas (2 studies), antithyroid drugs, hERG channel blockers, urinary antispasmodics, methadone, propoxyphene, any drug (1 study each).

**Fig. (2):**
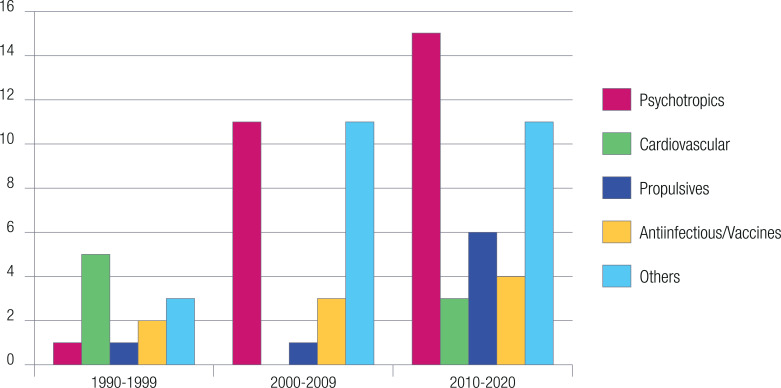
Drugs associated with different cardiovascular outcomes for decades.

We found out that there has been a change in the trend of the class of drugs studied: in the early 90s, cardiovascular drugs were in the spotlight, whereas the interest in antipsychotics and antidepressants has increased after 2000 and up until today (Fig. **2**).

#### 3.4.1 Antipsychotic Drugs

Four studies found an increased risk of different cardiovascular outcomes (SCD [27, 36, 76], a composite of CA/VA [37]) associated with the use of typical antipsychotics compared to non-use. One study found an increased risk of several cardiovascular outcomes (all-cause mortality, cardiac mortality, SCD) associated with antipsychotics (typical and atypical) compared to non-use [68]. Another study found an increased risk of SCD associated with the use of typical or atypical antipsychotics with no differences between them [48]. An increased risk of SCD at the time of an acute coronary event [62] or a composite of CA/VA was found for typical antipsychotics but not for atypical [37].

Some studies reported risks for individual drugs. An increased risk of a composite of VA/CA was found for haloperidol, thioridazine, clozapine or risperidone [31]. An increased risk of SD associated with the use of thioridazine [32] or clozapine [26] was also found in two other studies. Another one did not find any difference between ziprasidone and olanzapine regarding the risk of non-suicide mortality, SCD or SD [56, 59]. An increased risk for SCD and/or SD was found for quetiapine, olanzapine, risperidone, haloperidol, clozapine, and thioridazine [79]. Finally, an increased risk of a composite of SCD/VA was found for chlorpromazine or haloperidol compared to olanzapine; no difference was observed between olanzapine and risperidone, but quetiapine showed a lower risk compared to olanzapine [69].

#### 3.4.2 Antidepressants

One study [34] found an increased risk of SCD associated with the use of tricyclic antidepressants when used at ≥300 mg of amitriptyline or its equivalent but not when used at lower doses or when used at the time of an acute coronary event [61]. Another study also found an increased risk of out-of-hospital CA associated with tricyclic antidepressants [64]. Regarding selective serotonin reuptake inhibitors (SSRI), no increased risk of SCD was found in 2 studies [34,61] and in 1 of them when used at the time of an acute coronary event [61]. Another study found an increased risk of out-of-hospital CA associated with the use of SSRI; even though the lower 95% confidence interval was 1, an increased risk for citalopram was described [64]. This last study did not find an increased risk of out-of-hospital CA for serotonin-norepinephrine reuptake inhibitors/noradrenergic and specific serotonergic antidepressants [64].

Some studies reported no increased risks of SD for individual antidepressants, such as desipramine [23] or bupropion [39]. An increased risk of sudden CA [67] or out-of-hospital CA [64] associated with nortriptyline compared to no use was found in other studies. No increase in the risk of out-of-hospital CA was found for escitalopram, paroxetine, sertraline, imipramine, venlafaxine, mianserine or mirtazapine [64]. Regarding venlafaxine, it did not increase the risk of SCD compared to fluoxetine, citalopram or dosulepin [52]. Another study found an increased risk of a composite of SCD/VA for mirtazapine but not for other antidepressants compared to paroxetine [57]. Moreover, another study did not find an increased risk of a composite of SCD, other CV death and accidental overdose in patients treated with a high dose of citalopram or escitalopram compared to those treated with a high dose of other SSRI [82].

#### 3.4.3 Attention Deficit Hyperactivity Disorder Stimulants

Two studies did not find an increased risk of SD [47] or a composite of SD/VA [58] associated with stimulants in children, adolescents, and young adults. In contrast, 2 other studies did find an increased risk of sudden unexplained death, mainly driven by methylphenidate, in children and adolescents [45] or of a composite of SD/VA for methylphenidate in adults; however, the lack of a dose-response relationship did not support a causal relationship according to the authors [63].

#### 3.4.4 Cardiovascular Drugs

Four studies dealt with antiarrhythmics. One study found an increased risk of cardiac mortality and mortality due to arrhythmia in patients with atrial fibrillation and congestive heart failure treated with different drugs (quinidine, procainamide, disopyramide, flecainide, encainide, and amiodarone) [14]. Other studies reported a risk for individual agents. So, the risk of sudden death associated with the use of amiodarone was found to be higher among patients with advanced heart failure and torsade de pointes than among those without torsade de pointes [21] or the risk of cardiovascular mortality in patients with atrial fibrillation treated with flecainide was higher than in the general population [55]. An increased risk of SCD was associated with the use of digoxin in homozygous T allele carriers of ABC1 gene single-nucleotide polymorphisms C1236T, G2677T, and C3435T76.

Regarding antihypertensives, the 4 studies found an increased risk of different cardiovascular outcomes (out-of-hospital CA, CA or SCD) associated with the use of hypokalaemia-inducing antihypertensives *versus* users of antihypertensives with neutral potassium effect [81], *versus* hyperkalemia-inducing diuretics [19] or *versus* no use [15]. One of these studies also reported an increased risk of SCD among patients treated with β-blockers [19]. Another study identified an increased risk of CA in patients treated with high doses (100 mg) *versus* low doses (25 mg) of thiazides [17].

#### 3.4.5 Antiepileptic Drugs

One study did not find an increased risk of SUDEP in epileptic patients treated with antiepileptic drugs [65], but another study identified an increased risk of SCD associated with the use of antiepileptics as a group, the calcium channel blockers as a group or carbamazepine or gabapentin in symptomatic epileptic patients [73]. Other 3 studies found an increased risk of SUDEP in epileptic women treated with lamotrigine [66], in patients with high plasmatic levels of carbamazepine [29] or in patients treated with more than two antiepileptics [28]. Finally, a recent study has found that polytherapy (≥ 3 drugs) was associated with a substantially reduced risk, including combinations with lamotrigine, valproic acid, and levetiracetam [87].

#### 3.4.6 Propulsives

Six out of the 8 studies that dealt with domperidone found an increased risk of different outcomes, such as SCD [53, 72] or a composite of SCD/VA [51, 74, 77, 78]. The remaining studies referred to cisapride: on the one hand, a study found an increased risk of a composite of SCD/VA compared to proton pump inhibitors [44], while the other did not find a risk of a composite of death and cardiac death and VA compared to no use [25].

#### 3.4.7 Antibiotics

Four studies investigated the risk of different macrolides. Azithromycin was found to be associated with an increased risk of cardiovascular death and death from any cause compared to no use [63]. An increased risk of SCD was found for erythromycin [35]. The third study found an increased risk of a composite of CA/VA associated with the recent use of macrolides as a whole [50]. This latter study also found an increased risk of the same composite associated with fluoroquinolones [50]. One study showed an increased risk of SD associated with cotrimoxazol compared to amoxicillin in patients treated with spironolactone [71]. The fourth study did not find an increased risk of SD in digoxin-treated patients when erythromycin, azithromycin or clarithromycin was added compared to cefuroxime [83].

#### 3.4.8 Vaccines

Three studies did not find an increased risk of SIDS in children vaccinated with DTP [24], DTPP (diphtheria-tetanus-pertussis-poliomyelitis) [22] or DTPP±Hib (diphtheria-tetanus-pertussis-poliomyelitis +/– *Haemophilus influenza* type B) [30]. The fourth study only found an increased risk of SIDS in female children recently vaccinated with diphtheria, tetanus, and whole-cell pertussis vaccine [80].

#### 3.4.9 Non-cardiac QT-prolonging Drugs

Four studies dealt with non-cardiac QT-prolonging drugs. One included several drugs and found an increased risk of CA in patients treated with domperidone, haloperidol, and cotrimoxazole [42]. Another study, also including several non-cardiac QT-prolonging drugs, found an increased risk of SCD associated with domperidone and haloperidol [38]. The third study involved the drugs included in the Arizona classification and found an increased risk of SD for atypical and typical antipsychotics as well as for SSRI as a whole and for risperidone, fluoxetine, and sertraline as individual drugs [46]. A fourth study reported an increased risk of SCD for QT-prolonging medications in low and high exposure groups, as well as in non-sudden arrhythmic death [86].

#### 3.4.10 COX-2 Inhibitors

Two studies evaluated the risk of NSAIDs and/or COX-2 inhibitors. One study found an increased risk of SCD and MI variable composite for rofecoxib and celecoxib [40], whereas another study found an increased risk of a combined variable of the acute coronary syndrome and SCD for rofecoxib, but not for celecoxib or naproxen [41].

#### 3.4.11 Antihistamines

Two studies did not find an increased risk of VA or a composite of SD/VA/QTc prolongation in patients treated with terfenadine compared to other antihistamines [16,18]. However, both studies found an increased risk of the corresponding outcome in patients concomitantly treated with ketoconazole [16] or with erythromycin [18]. A third study did not find an increased risk of a composite of SD/VA associated with the use of astemizole compared to other sedating antihistamines [20].

#### 3.4.12 Second-generation Sulfonylureas

Two studies did not find an increased risk of a composite of SCD and VA in patients treated with second-generation glimepiride and glyburide compared to glipizide [84, 85].

## 4 DISCUSSION

To our knowledge, this is the first scoping review that analyses the risk of the drugs related to sudden death and the methodology used. We identified 74 studies, being the first cohort study assessing the risk of cardiac death in patients with AF treated with antiarrhythmic drugs [14].

The number of studies has increased over time, and studies have been carried out fundamentally in Europe and the United States, being case-control and cohort the most frequently used study design. We have not found any ad-hoc observational study; most have used information contained in databases, being the administrative ones more used than epidemiological ones. Few studies have studied SCD as a single outcome. The majority used a combined variable that included SCD or SD as well as other variables, based on the information contained in databases along with death certificate information or medical history in some cases. Five studies analyzed the risk of SUDEP and another 4 for SIDS. In very few studies, the outcome was validated by an expert, and the result of a confirmation autopsy was provided. Even though they are considered different diseases with a common outcome, specific populations, such as SUDEP and SUID, were included as the studies evaluated mortality due to drug use.

In general, the studies dealt with specific drugs or groups of drugs. Antiarrhythmics, diuretics, and antihistamines were the drugs most frequently studied in the 90s; in contrast, antipsychotics, antidepressants, antibiotics vaccines, and propulsives were mostly researched in the following decades. There are many other drugs investigated, but a few studies are conducted on each. Only 3 studies aimed at assessing a group of drugs selected from the effect on the QT interval.

In relation to antipsychotics, at least 4 studies coincide in identifying an increased risk of various outcomes associated with the use of typical antipsychotics. However, the results of studies carried out with atypical antipsychotics are conflicting. Tricyclic antidepressants have been associated with an increased risk of different result variables in 3 studies, even though in one of them, this effect was just observed in patients treated with high doses and in patients with an acute coronary event in another study. Regarding SSRI, results are contradictory, although they aim not to increase the risk of a fatal cardiac outcome. Furthermore, as far as attention deficit hyperactivity disorder stimulants are concerned, the results of the studies are contradictory too. In general, antiarrhythmic drugs and hypokalemia-inducing antihypertensives showed an increased risk of several outcomes. Studies on propulsive drugs included predominantly domperidone, which has shown an increased risk of death in all the studies carried out. Even though only 3 studies published were found, macrolides were the most frequent antibiotics studied, and the results suggested an increased risk for the group as a whole or erythromycin and azithromycin. Similarly, 2 studies dealing with coxibs suggested an increased risk for rofecoxib. Regarding antiepileptics and SUDEP, results are contradictory, as older studies showed an increased risk in some subgroups of patients, and a recent study has shown a protective effect. Finally, the DTP vaccine did not seem to increase the risk of SIDS.

For the majority of drugs studied, the biological plausibility of the association is based on the interference on the heart depolarization and repolarization by a combination of the blockage of sodium, calcium, and potassium ion channels present in the ventricular myocytes, and therefore modifying QTc interval [7], which is manifested electrocardiographically by an excessive QT prolongation that can provoke a torsade de pointes arrhythmia and SD. Given the seriousness of this adverse drug reaction, the International Registry for Drug-Induced Arrhythmias Arizona (Arizona Centre for Education and Research on Therapeutics, http://www.dpic.org/links/arizona-center-education-and-research-therapeutics) was created, which collects and classifies drugs in various categories according to their ability to cause QT interval prolongation. Although the majority of patients who die from sudden arrhythmogenic cardiac death do so as a result of ventricular fibrillation, evidence shows that non-defibrillating rhythms are increasing [88-90], such as extreme bradycardia or asystole. Drugs with a positive chronotropic effect have also been implicated as SCD causes. In addition, some medications lack a direct arrhythmogenic effect but can cause hydroelectrolytic abnormalities, such as hypocalcaemia, hypomagnesaemia, hypokalemia, some medications and hyperkalemia, which can trigger fatal ventricular arrhythmias. Except for hypokalemic diuretics, for which we have found some studies, the information available for these drugs is scarce or practically nil.

The methodologies used, especially regarding administrative databases and also the other sources of information, present serious limitations when it comes to truthfully identifying the cause of death, which may jeopardize the validity of the results. This limitation is especially noteworthy when the study disease is SCD, which is frequently misdiagnosed and misclassified in such information sources. This is because its occurrence is frequent outside medical settings and lacks witnesses. For this reason, studies that have investigated etiological factors of sudden cardiac death based on records, such as the death certificate, have low validity: in such cases, the use of necropsy diagnosis is recommended to rule out non-cardiac causes or other causes of cardiac etiologies [91].

More recently, the validation of a computer case definition of SCD has been published based on the information contained in an administrative database, together with the information from the death certificate and the hospital discharge database. However, 45% of the sample cases could not be validated due to a lack of information [92].

No studies, evaluating the correlation between SD and certain types of drugs that are extensively used in determined populations, such as chemotherapy, monoclonal antibodies, tyrosine-kinase inhibitors or antiretroviral drugs, were found. Given the increase in survival of these patients due to their treatment, SD should be evaluated in these populations. Moreover, no studies assessing the risk of drug-induced SD in populations, such as chronic kidney disease or hepatic insufficiency, were found.

Recent concerns have been raised regarding the impact of water [93, 94] and air [95] pollution on human health, as demonstrated by Bañeras *et al*., who showed an increase in myocardial infarction incidence and mortality regarding higher air pollution levels. However, contamination was not recorded nor evaluated in the studies included in this scoping review.

At present, operative registries of SD in young patients or athletes are available. Even though SD secondary to drug use diagnosis is difficult and requires autopsy to exclude other causes, its attribution can never be completely accurate, and SD related to drug use database could be considered. Finally, it should be noted that only a quarter of the studies carried out used the variable SCD. The most commonly used outcome is a combined variable that, among other limitations, makes it difficult or impossible to know the real risk of SCD [96].

### 4.1 Limitations

Our scoping review also has some limitations that should be taken into account. Firstly, SUDEP and SIDS terms were not included in the research strategies and, therefore, might be underrepresented in this scoping review. Both search strategies contributed to approximately 75% of the total articles found, while 25% were found after reference review, suggesting that article indexing included some terms that were not included in our search. In order to improve our search strategies, MeSH terms were reviewed, and it was found that terms, such as Sudden Death, were not found in some studies even though evaluating this outcome. Moreover, research was only carried out using the Medline database and included studies in English, so some studies available in other databases or other languages may not have been identified. Finally, due to the nature of a scoping review, a critical appraisal of the studies included was not performed after consensus between the main investigators.

## CONCLUSION

Drugs most frequently associated with SD are antipsychotics, antidepressants, and propulsive. Study designs are heterogeneous, but predominantly observational studies were performed. The main source of information was medical databases. Only 18.9% of the studies included autopsy results to confirm the death. Studies combining preferably epidemiological databases with other sources of information as well as autopsy results should be recommended in order to assess the risks associated with drug use and confirm the outcome.

## Figures and Tables

**Fig. (1):**
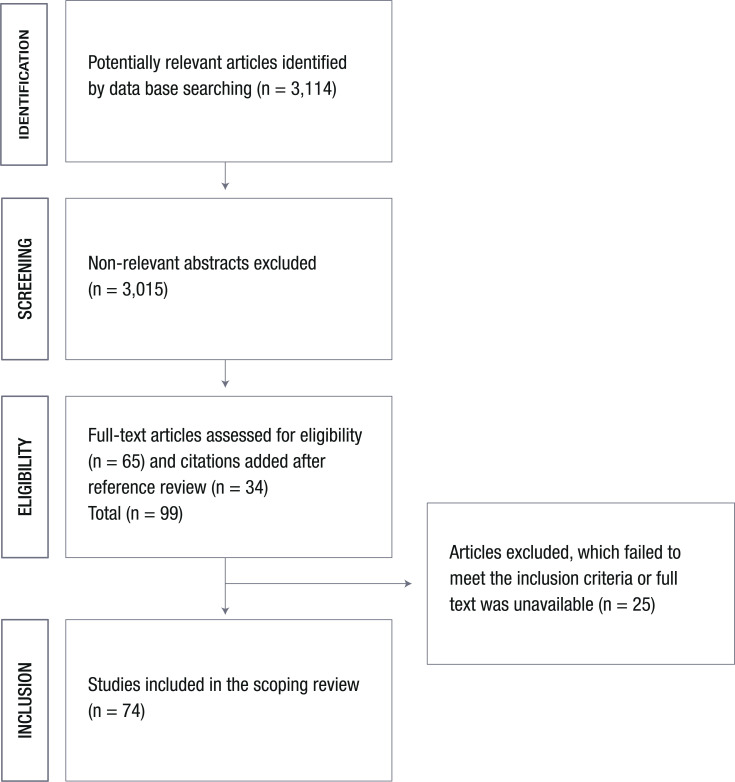
PRISMA flow chart of the studies included in the scoping review.

**Table 1:** Main characteristics of included studies.

**-**	**1990 – 1999** **(n = 12)**		**2000 – 2009** **(n = 25)**		**2010 – 2020** **(n = 37)**		**Total** **(n = 74)**	
**-**	**n**	**%**	**n**	**%**	**n**	**%**	**n**	**%**
**Geographical area** USA Europe Canada Other Not pertinent	7 3 1 2	58.3 25.0 8.3 16.7	13 11 1	52.0 44.0 4.0	10 15 4 4 4	27.0 40.5 10.8 10.8 10.8	30 29 5 5 6	40.5 39.2 6.8 6.8 8.1
**Design** Case control Cohort Systematic review / meta-analysis Other	3 6 2 1	25.0 50.0 16.7 8.3	13 12 4	52.0 48.0 16.0	19 12 3 5	51.4 32.4 8.1 13.5	35 30 5 10	47.3 40.5 6.8 13.5
**Source of outcome data** Administrative database Epidemiological database Medical chart / Hospital discharge report Death certificate Autopsies Patient’s registry Mortality registry Contact with physician and/or relatives Other	5 1 2 1 2 2 1 1 3	41.7 8.3 16.7 8.3 16.7 16.7 8.3 8.3 25.0	11 6 10 6 7 3 4 3 1	44.0 24.0 40.0 24.0 28.0 12.0 16.0 12.0 4.0	12 7 12 8 5 6 6 1 4	32.4 18.9 32.4 21.6 13.5 16.2 16.2 2.7 10.8	28 14 24 15 14 11 11 5 8	37.8 18.9 32.4 20.3 18.9 14.9 14.9 6.8 10.8
**Outcome** Composite variable SCD SD Mortality by any cause CA SUDEP SIDS Cardiovascular mortality Cardiac mortality VA	3 2 2 1 2 1 1	25.0 16.7 16.7 8.3 16.7 8.3 8.3	6 8 7 1 2 1	24.0 32.0 28.0 4.0 8.0 4.0	17 8 5 5 4 4 1 3 1	45.9 21.6 13.5 13.5 10.8 10.8 2.7 8.1 2.7	26 18 14 6 5 6 4 3 2 1	35.1 24.3 18.9 8.1 6.8 8.1 5.4 4.1 2.7 1.4
**Source of exposure data** Prescription data Dispensing data Clinical trial Contact with relatives Toxicology at autopsy Other	5 5 1 3	41.7 41.7 8.3 25.0	17 6 2 1	68.0 24.0 8.0 4.0	22 6 2 1 5	59.5 16.2 5.4 2.7 13.5	44 17 3 3 1 8	59.5 23.0 4.1 4.1 1.4 10.8
**Control of confounding** Adjusted analysis Unadjusted analysis Other ^a^	7 3 2	58.3 25.0 16.7	21 4	84.0 16.0	30 4 3	81.1 10.8 8.1	58 11 5	78.4 14.9 6.8

**Note:** ^a^Meta-analysis, systematic reviews

**Abbreviations:** CA: cardiac arrest; SCD: sudden cardiac death; SD: sudden death; SIDS: sudden infant death syndrome; SUDEP: sudden unexpected death in epilepsy.

## LIST OF ABBREVIATIONS

SCD: Sudden Cardiac Death
DTP: Diphtheria-Tetanus-Pertussis
CA: Cardiac Arrest
SUDEP: Sudden Unexpected Death in Epilepsy
SIDS: Sudden Infant Death Syndrome
DTPP: Diphtheria-Tetanus-Pertussis-Poliomyelitis

## SUPPLEMENTARY MATERIAL

PRISMA checklist is available as supplementary material on the publisher’s website along with the published article.
